# RV dysfunction by MRI is associated with elevated transpulmonary gradient and poor prognosis in patients with sickle cell associated pulmonary hypertension

**DOI:** 10.1186/1532-429X-15-S1-O43

**Published:** 2013-01-30

**Authors:** Kim-Lien L Nguyen, Shoaib Alam, Xin Tian, Steve W Leung, Catherine Seamon, Caterina P Minniti, James G Taylor, Vandana Sachdev, Andrew E Arai, Gregory J Kato

**Affiliations:** 1 Cardiovascular and Pulmonary Branch, National Heart, Lung, and Blood Institute, Bethesda, MD, USA; 2 Sickle Cell Vascular Disease Section, Hematology Branch, National Heart, Lung, and Blood Institute, Bethesda, MD, USA; 3 Office of Biostatistics Research, National Heart, Lung, and Blood Institute, Bethesda, MD, USA; 4 Division of Cardiovascular Medicine, University of Kentucky, Lexington, KY, USA

## Background

Patients with sickle cell disease (SCD) and pulmonary hypertension (PH) have increased mortality. SCD-PH is often complicated by high cardiac output (CO) related to anemia. The transpulmonary gradient (TPG) reflects a pressure differential across the pulmonary vascular bed without the confounding effect of CO (PVR=TPG/CO). Based on the cardiac transplant literature, a TPG ≥ 12 mmHg indicates significant pulmonary arterial hypertension (PAH). With PH, there is often morphologic adaptation by the right ventricle (RV). In idiopathic PAH, RV dilation and decreased function have been correlated with poor prognosis. We hypothesize that patients with SCD and a TPG ≥ 12 mmHg would have lower functional capacity, increased mortality, and evidence of RV dysfunction on cardiac MRI (CMR).

## Methods

Five hundred and twenty nine consecutive patients (age 35.5 ± 12.5, 54% (n=283) female, 73% (n=387) HbSS) with SCD were prospectively screened for PH using echocardiography (tricuspid regurgitant jet ≥ 2.5 m/s) without any exclusion criteria. Eighty four (age 41 ± 13, 55% (n=46) female, 82% (n=69) HbSS) underwent right heart catheterization (RHC), and 41 (age 42 ± 15, 54% (n=22) female, 80% (n=33) HbSS) underwent CMR within one week of RHC. CMR sequences consisted of cine and late gadolinium enhancement imaging.

## Results

Those with a TPG ≥ 12 mmHg had higher mortality (p=0.008), poorer functional class (p=0.007), shorter 6-minute walk distance (p=0.003), and lower cardiac index (p=0.001). High TPG patients demonstrated more abnormal CMR markers of RV dysfunction (lower tricuspid annular plane excursion (p=0.001) and RV ejection fraction (p=0.002)). Recently validated CMR markers of PAH including septal-to-LV-free-wall curvature ratio (p=0.013), septomarginal trabeculae mass index (p=0.002), and ventricular mass index ratio (p=0.016) were significantly different in the high TPG group. Left ventricular (LV) eccentricity index (EI) at end-systole (ES) was greater in the high TPG group (p=0.026) without a significant change between ES and end-diastole (ED)—consistent with RV pressure overload. In the low TPG group, the EI-ED was greater than EI-ES (p=0.001)—consistent with RV volume overload. LV ejection fraction was similar in both groups. Late gadolinium enhancement at the RV insertion points indicative of myocardial fibrosis occurred more frequently with higher TPG (p=0.030).

## Conclusions

CMR evidence of RV dysfunction was associated with a TPG ≥ 12 mmHg. A TPG ≥ 12 mmHg effectively identified patients with a lower functional capacity and overall worse prognosis. The TPG, being less subject to the confounding effects of CO, further demonstrates the functional severity of pulmonary arterial disease in SCD using objective thresholds established in the cardiac transplant literature.

## Funding

None.

